# Sacroiliitis mimics: a case report and review of the literature

**DOI:** 10.1186/s12891-017-1525-1

**Published:** 2017-04-22

**Authors:** Maria J. Antonelli, Marina Magrey

**Affiliations:** Case Western Reserve University, MetroHealth Medical Center, Divison of Rheumatology, 2500 MetroHealth Dr., Cleveland, OH 44109 USA

**Keywords:** Case report, Ankylosing spondylitis, Clinical diagnostics & imaging, Rheumatic disease

## Abstract

**Background:**

Radiographic sacroiliitis is the hallmark of ankylosing spondylitis (AS), and detection of acute sacroiliitis is pivotal for early diagnosis of AS. Although radiographic sacroiliitis is a distinguishing feature of AS, sacroiliitis can be seen in a variety of other disease entities.

**Case presentation:**

We present an interesting case of sacroiliitis in a patient with Paget disease; the patient presented with inflammatory back pain which was treated with bisphosphonate. This case demonstrates comorbidity with Paget disease and possible ankylosing spondylitis. We also present a review of the literature for other cases of Paget involvement of the sacroiliac joint.

**Conclusions:**

In addition, we review radiographic changes to the sacroiliac joint in classical ankylosing spondylitis as well as other common diseases. We compare and contrast features of other diseases that mimic sacroiliitis on a pelvic radiograph including Paget disease, osteitis condensans ilii, diffuse idiopathic skeletal hyperostosis, infections and sarcoid sacroiliitis. There are some features in the pelvic radiographic findings which help distinguish among mimics, however, one must also rely heavily on extra-pelvic radiographic lesions. In addition to the clinical presentation, various nuances may incline a clinician to the correct diagnosis; rheumatologists should be familiar with the imaging differences among these diseases and classic spondylitis findings.

**Electronic supplementary material:**

The online version of this article (doi:10.1186/s12891-017-1525-1) contains supplementary material, which is available to authorized users.

## Background

The presence of sacroiliitis on an anterior-posterior (AP) pelvis or dedicated sacroiliac film is a defining feature of ankylosing spondylitis (AS). Sacroiliac (SI) joint abnormalities consistent with sacroiliitis include subchondral sclerosis, uniform joint space narrowing and erosions in early to advanced disease, with later progression to ankylosis and obliteration of the SI joint [1]. Radiographic changes of sacroiliitis are not only included in the 1984 New York Classification Criteria for AS [2] but also in the new classification criteria established by the Assessment of SpondyloArthritis international Society (ASAS classification criteria) [3]. Although radiographic sacroiliitis is a distinctive feature of AS, findings of sacroiliitis can be seen in a variety of other disease entities. Here, we present an interesting case of sacroiliitis in a patient with Paget disease. Our aim is to review other pathologies that mimic sacroiliitis on a pelvic radiograph.

We conducted a search in PubMed including combinations of the following search terms: sacroiliitis, sacroiliac, and Paget disease. We filtered for case reports and case series. Inappropriate and unobtainable publications were omitted. Two publications (published in 1999 and 2010) and seven cases were identified and used in this discussion (described in Table 3).

## Case presentation

A 54 year-old African-American male with long-standing back pain presented to the rheumatology clinic for evaluation. He was healthy except for chronic hypertension, hyperlipidemia, depression, and chronic back pain. He had no prior surgeries and did not smoke tobacco or drink alcohol. Family history was negative. History of back pain dated back 20 years. Over the past ten years, he had been treated with non-steroidal anti-inflammatory medications which he took for several weeks at a time when his pain flared. In the last three months, his back pain had been worsening, now with deep left gluteal pain. The patient denied any trauma to the area or recent falls. He initially had relief with oral diclofenac 75 mg twice daily but in the last two months had had pain which was not controlled on that dose. In addition he noted limited mobility. He reported morning stiffness for 30 min daily. His pain did not improve with exercise. He denied eye redness or pain, sexually-transmitted infections, chronic diarrhea or bloody stools, psoriasis, enthesitis- or dactylitis-like episodes presently and in the past.

On examination, his heart rate was 59 beats per minute and regular, his respiratory rate was 12, and blood pressure was 158/94. He was generally comfortable and well-developed and well-nourished. His cardiac and pulmonary examination was within normal limits. Joint examination revealed no synovitis, enthesitis or dactylitis. His occiput to wall measurement was zero. There was some thoracic spinal and paraspinal tenderness with excruciating left SI tenderness to palpation. The Schober maneuver exhibited more than 5 cm of excursion. The FABER exam elicited pain in bilateral SI joints. Neurologic exam revealed no sensory deficits, normal motor strength, and normal deep-tendon reflexes.

**Fig. 1:**
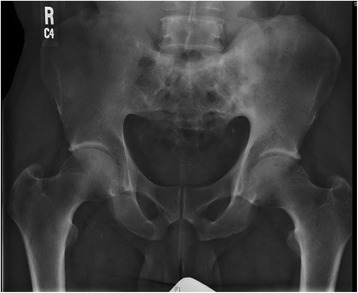
Pelvic X-ray on presentation. The patient’s pelvic film demonstrates thickened trabeculae and sclerosis in addition to bony ankylosis of sacroiliac joints bilaterally. The trabecular pattern appears to partially extend into the *left medial iliac region*

Available laboratory data at the time of presentation are summarized in Table 1. His complete blood count, complete metabolic panel and inflammatory markers were all within the reference ranges except for a mild anemia which had been long present, as well as elevated sedimentation rate. A previous X-ray of the pelvis from nine years earlier reported coarsened trabeculae with sclerosis of the sacrum and left ilium with ankylosis of SI joints (film unavailable). The current pelvic film (Fig. 1) showed thickened trabeculae and sclerosis in addition to bony ankylosis of sacroiliac joints bilaterally. The trabecular pattern appeared to partially extend into the left medial iliac region.

**Table 1:** Presentation Laboratory Values

Laboratory Parameter	Patient laboratory value at presentation	Reference Range
Hemoglobin (g/L)	123	140–175
WBC count (x10^9^/L)	6.0	4.5–11.5
Platelets (x10^9^/L)	197	150–350
Creatinine (μmol/L)	61	30–120
Alkaline Phosphatase (μkat/L)	3.31	0.5–2.0
Erythrocyte sedimentation rate (mm/h)	50	<20
C-reactive protein (nmol/L)	<4.76	0.76–28.5
HLA-B-27	Negative	-

Available laboratory data at the time of presentation are summarized here. The patient’s complete blood count, complete metabolic panel and inflammatory markers were all within reference range except for a mild anemia which had been long present as well as an elevated sedimentation rate

**Fig. 2:**
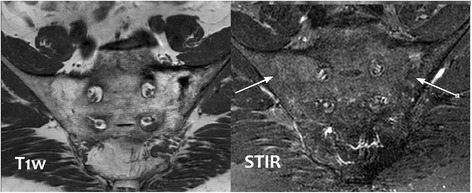
MRI of bony pelvis. The MRI of the patient’s pelvis reveals bony fusion of the bilateral sacroiliac joints with thickening of the cortex of the *left iliac bone* and bony trabeculation of the sacrum and *left iliac bone*. Degenerative changes are seen in the visualized *lower lumbar spine*. There is a hyperintense signal intensity in the *left sacral ala* which is likely due to micro trabecular insufficiency fractures

The patient’s pain was thought to be due to Paget disease and he was treated with zoledronic acid. His pelvic pain improved with the treatment. Six months after the infusion of zoledronic acid, however, back pain recurred. An MRI was done to further delineate the cause of the pain. The MRI of the pelvis revealed bony fusion of the bilateral sacroiliac joints with thickening of the cortex of the left iliac bone and bony trabeculation of the sacrum and left iliac bone (Fig. 2). Degenerative changes were seen in the visualized lower lumbar spine. There was a hyperintense signal in the left sacral ala which was likely due to micro-trabecular insufficiency fractures. A timeline of the patient’s presentation and findings can be found in the Case Timeline Additional file 1.

## Discussion and conclusions

The superior-posterior portion of the SI joint, a bony cleft (without cartilage or synovium) joined by ligaments, lies in a horizontal-oblique orientation. The anterior-inferior portion of the SI joint, a true synovial joint, lies vertically oriented [4, 5]. The cartilage on the iliac side of the synovial part of the SI joint is thinner; thus, all SI disease processes involve the iliac side before the sacral side [5]. The location and the characteristics of the pathologic changes often give a clue to etiology. The main radiographic signs of sacroiliitis are bone erosions, joint space alterations, subchondral sclerosis, and ankylosis [1, 5]. Sacroiliitis from spondyloarthritis, enteropathic arthropathy and osteitis condensans ilii is usually bilateral and symmetric, but unilateral and asymmetric changes may also be seen, particularly in psoriatic arthritis and reactive arthritis, generally early in disease [5]. These SI joint changes are in contrast to degenerative features of the SI joint which include osteophytes, dense sclerosis mainly on the iliac side, and joint space narrowing [6].

Vertebral manifestations of AS present later in disease and include vertebral “ivory corner” of sclerosis with progression to syndesmophytes [5]. MRI findings during acute sacroiliitis include intra-articular fluid, subchondral bone marrow edema, articular and periarticular post-gadolinium enhancement, and soft tissue edema. Findings of chronic disease include periarticular bone marrow conversion, bone erosion, subchondral sclerosis and ankylosis [4]. Erosions are best demonstrated on T1- weighted fat-saturated sequences as high intensity signals. Subchondral bone marrow edema is characterized by low-intensity signal on T1 and high-intensity on T2 and STIR sequences [7]. Sacroiliitis mimics can be seen in other disease entities as discussed below. A table of comparative features of these mimics is depicted in Table 2.

**Table 2:** Features of AS/Spondylitis Sacroiliitis and Disease Mimics

	Spondylitis & AS	Paget Disease of bone	DISH	Osteitis Condensans Ilii	Sarcoidosis	Brucella & other infections
Pelvic Radiographic findings	-Symmetric, bilateral changes (AS) -unilateral changes more common with other spondylitis -primarily inferior (synovial) part of the joint -erosions present -ankylosis at end stage	-iliac wing, iliopectineal and ischiopubic lines: cortical thickening and sclerosis -lytic & sclerotic lesions -mostly asymmetric presentation	-primarily superior (ligamentous) joint area -appearance of “ankylosis”	-triangle of sclerosis in the ilium adjacent to the inferior SI joint -often bilateral -no erosions -normal joint space (no narrowing)	-mostly unilateral, but can be bilateral -sclerosis and irregularities of sacroiliac joints margins -may involve synovial or cartilaginous parts, dependent on if boney or joint granulomatous infiltration	-no Xray changes until about 15 days of infection -extensive erosions -subsequent boney repair (may involve more than the anterior-inferior part) which may progress to ankylosis
Extra-pelvic radiographic lesions	-continuous spine involvement (no skip lesions) -shiny corner on vertebra bodies involved; squaring of vertebral bodies	-skull and long bones are commonly involved	-Skip lesions in the spine -bulky osteophytic bridging and ossification -“flowing” ALL ossification -all spinal segments, but typically thoracic -extra-spinal entheseal ossification, periarticular hyperostosis of the hands, knees, and elbows and quadriceps tendon	-none	-Often hilar lymphadenopathy, infiltrates on CXR -osseous findings such as reticular “lacy” pattern in phalanges	-Dependent on infectious localization
Genetic Association	HLA-B27	HLA-DR2, *SQSTM1* gene	Possibly some SNPs in *COL6A1*	No HLA association	*BTNL2* gene	HLA-B39 (*Brucellosis)*
Age at onset, Gender	<40 years old, likely equal sexes	>40 years old	>50 years old	Middle-age, predominantly multiparous females <40 years old	Middle-age	Any age (typically young adults and children)

As depicted here, there are some features in the pelvic radiographic findings which help distinguish among sacroiliitis mimics. There is also heavy reliance on extra-pelvic radiographic lesions

### Paget disease

Paget disease, also known as osteitis deformans, is a common bone disease that includes lytic, blastic and mixed phases of bony changes [8]. There is familial and geographic population clustering of the disease, suggesting a possible genetic component, with some increased frequency in HLA-DR2 [8] and *SQSTM1* gene [9], as well as an environmental component to the disease. The skull and long bones are commonly involved as well as the pelvis. The iliac wing, iliopectineal, and ischiopubic lines can show cortical thickening and sclerosis, with mostly asymmetric presentation [8].

Fusion of SI joints can be seen in Paget disease either unilaterally or bilaterally [10]. In our review of the literature, we identified seven cases of Paget disease involvement of the sacroiliac joint in two different case series (Table 3). Six of the cases [10] were confirmed on computed tomography of the pelvis: demonstration of cortical and trabecular thickening as well as areas of lysis. The seventh case was reported in a case series regarding Paget disease in Saudi Arabia [11]. This male patient presented with sacroiliac pain with normal laboratory findings. His radiograph showed sclerotic lesions in the left ilium (in addition to pubis, ischium and fifth lumbar vertebra). A bone scan revealed increased uptake in the areas, concerning for bony metastases. However, a bone biopsy showed typical bone changes of Paget disease. It was not until three years later that his alkaline phosphatase increased, with worsening pain. The patient’s sacroiliac joint pain improved with daily alendronate therapy.

**Table 3:** Paget cases in the literature with sacroiliac involvement

Case	Sex	Age	Alkaline Phosphatase (IU/L)	Laterality	Imaging	Confirmation of Paget disease	Comments
Case 1 [10]	Female	93	80	Bilateral	Right SI fusion	CT confirmation	Chondrocalcinosis present
Case 2 [10]	Female	81	120	Bilateral	Left SI fusion	CT confirmation	
Case 3 [10]	Male	70	160	Left unilateral	Left SI fusion	CT confirmation	
Case 4 [10]	Female	86	70	Bilateral	Bilateral fusion	CT confirmation	Chondrocalcinosis present
Case 5 [10]	Male	70	338	Bilateral	Bilateral fusion	CT confirmation	Comorbid ankylosing spondylitis
Case 6 [10]	Female	76	80	Right unilateral	No fusion	CT confirmation	
Case 7 [11]	Male	45	106	Left unilateral	Plain x-ray: sclerotic lesions in the left ileum, pubis, ischium and the fifth lumbar vertebra Bone scan: increased uptake same areas	Bone biopsy with Pagetoid changes	

This table depicts the features of the published cases of Paget involvement of the sacroiliac joint. Two publications (published in 1999 and 2010) and seven cases were identified and used in this discussion

Paget disease lesions typically do not extend across healthy joints, suggesting that when both SI joints are involved, one might suspect inflammatory or other joint damage prior to pagetoid involvement [9, 12]. Hence, right SI joint involvement in our patient raises the question of possible AS as no pagetoid changes were seen on that side. Co-morbid AS and Paget disease is unusual, but cited throughout the literature [10, 12, 13]. One prior case of documented Paget disease in a patient with AS suggested the use of Amor’s criteria in making the AS diagnosis, as those criteria do not require radiographic sacroiliitis for diagnosis [10]. In addition, lone sacroiliac involvement is an uncommon presentation, seen in only approximately 10% of AS cases, compared to the majority of cases in which pelvic with ascending spinal involvement occur [14].

**Fig. 3:**
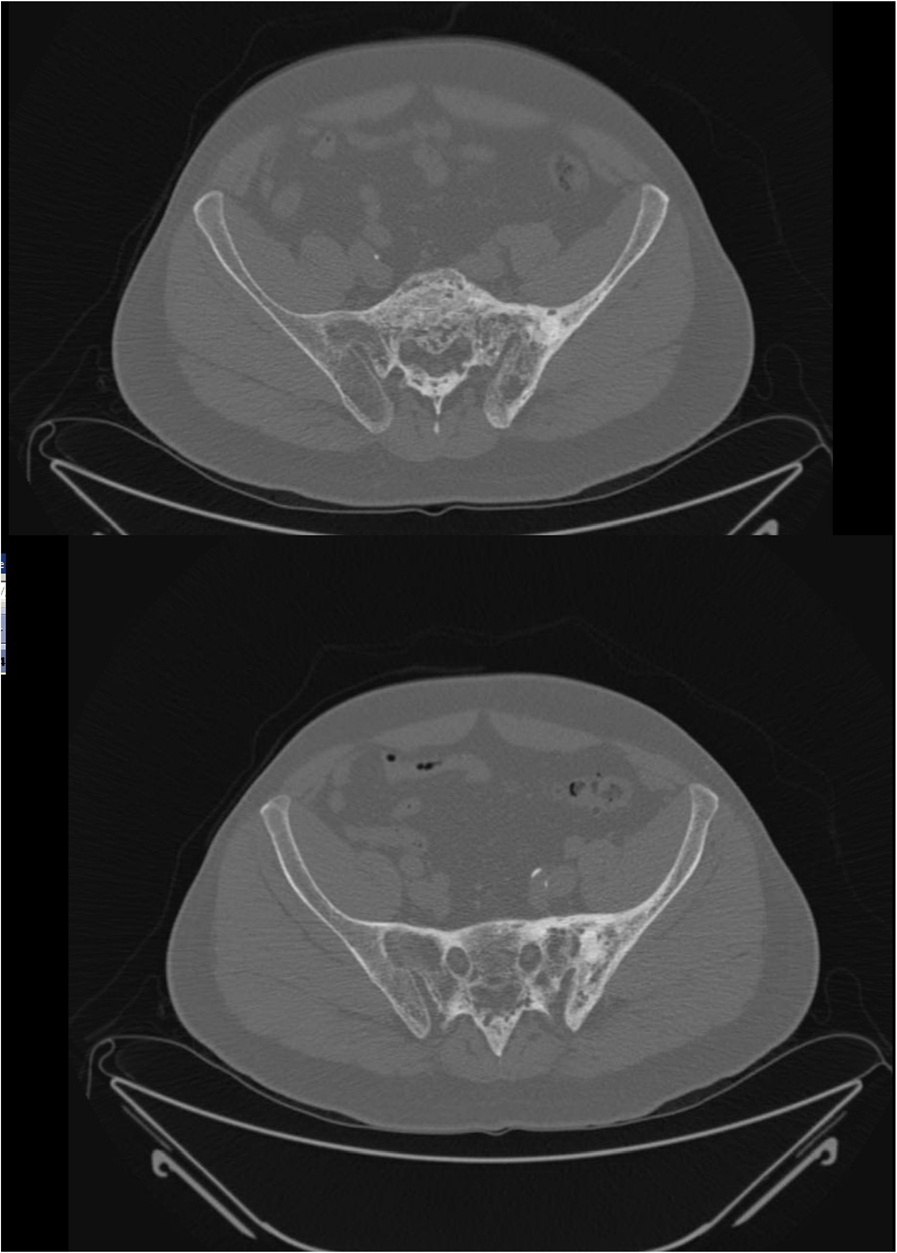
CTscan of pelvis. A CT scan of the pelvis of the patient reveals that the fusion of the SI joint is superior-posteriorly and the synovial part of the joint is patent. This observation suggests that involvement of SI joint can be seen without any pagetoid changes on that side

Our patient has definite Paget disease of the left sacrum and ilium with bilateral SI joint fusion and sclerosis. However, involvement was also seen in the right SI joint even though the right sacrum and ilium were not affected by Paget disease. In addition, his long-standing history of back pain from a young age brings to question prior or current spondylitis. For our case, a CT scan of the pelvis revealed that the fusion of the SI joint was superior-posterior and the synovial part of the joint was patent as shown in Fig. 3. This observation suggests that involvement of the SI joint can be seen without any pagetoid changes on the same side.

### Sacroiliitis and Diffuse Idiopathic Skeletal Hyperostosis (DISH)

Diffuse Idiopathic Skeletal Hyperostosis (DISH) is a systemic disease in which diffuse calcification involves ligaments, entheses and soft tissues [15]. The resultant clinical presentation may include impaired spinal mobility and postural abnormalities which may be misidentified as ankylosing spondylitis. Many terms have been used in the literature to describe this disease entity. It was officially named as early as 1950 by Forestier and Rotes-Querol; however, the disease has been recognized since ancient times [16]. This disease is diagnosed using various radiographic criteria, most commonly the Resnick diagnostic criteria which depend on four “flowing,” coarse osteophytes that bridge contiguous thoracic vertebra as depicted on a lateral film [17]. It has been postulated that metabolic factors may be involved in the pathogenesis of this disease, including the Wnt-β-catenin pathway, well known in bone physiology [16]. There are some data supporting a genetic component to DISH, possibly involving single nucleotide polymorphisms (SNPs) in the *COL6A1* gene [16]. Some debate has ensued as to whether there is clinical significance to DISH [15, 16].

Involvement of the sacroiliac joints in DISH can mislead physicians to believe that a patient has sacroiliitis. DISH imaging findings of the SI joint are not truly “sacroiliitis.” The upper portion of the SI joint is ligamentous and not a synovial joint as the bottom one-third of the joint is. Thus, with diffuse calcification and ossification, radiographs may show vacuum phenomena, narrowing sclerosis, and partial to complete ankylosis. In addition, the anterior capsule of the synovial part of the SI joint can show “ankylosis,” in which the anterior surface may become ossified [15]. The pelvis radiograph of a DISH patient may mistakenly be interpreted as “complete ankylosis” or “complete fusion” of the sacroiliac joint while close inspection and adjacent evidence might indicate otherwise. This deceptive nuance can be delineated with computed tomography (CT), in which the joint space can be better evaluated. In patients with DISH, the joint space is maintained without erosions [15]. DISH can also involve other parts of the axial spine, typically the thoracic spine. Bulky osteophytes bridge and give a “flowing” pattern to the anterior longitudinal ligament (ALL) ossification. Importantly, DISH is not limited to the axial skeleton: extra-spinal entheseal ossification, periarticular hyperostosis of the hands, knees, and elbows, and quadriceps tendon insertion are often found in DISH. These changes can be helpful in differentiating DISH from AS and other spondylitides.

### Osteitis condensans ilii

Osteitis Condensans Ilii (OCI) is another common sacroiliitis mimic affecting 0.9 to 2.5% of the general population [18]. With a predilection for multiparous females, the etiology of OCI has been postulated to be related to the physiologic changes of pregnancy, either the result of vascular compression with resultant ischemia, or mechanical laxity and sacroiliac joint overload. Nevertheless, OCI does afflict nulliparous females as well as males [18, 19]. Histopathologically, there is a quantitative increase in lamellar bone in the OCI lesions [18].

Axial low back pain more than back stiffness is the predominant symptom in OCI [19], and pain may be intermittent with sclerotomal radiation [18]. There are no sensory or motor changes on physical exam [18]. When pain in this site is found during pregnancy, it is generally in the third trimester or immediately after delivery, but may recur with subsequent pregnancies [18]. OCI may also present with chronic back pain and sometimes ‘hip’ pain. In addition, this entity may be noted radiographically but be clinically asymptomatic [20]. Other clues that may suggest a diagnosis of OCI are lack of systemic inflammation and findings: no weight loss, no anemia, normal inflammatory markers, and no eye inflammation or enthesophytes [19]. Additionally, OCI has not been associated with any particular HLA antigens [18].

Radiographically, OCI is seen with an apparent triangle of sclerosis in the ilium contiguous to the inferior SI joint–there are no erosions or joint space narrowing [21]. Although predominant iliac involvement is likely the namesake origin for OCI, sacral sclerosis is seen in OCI as well [18, 22]. OCI lesions are typically bilateral and symmetric [18], but may be unilateral [19]. MRI will demonstrate sclerosis of the ilium, possibly the adjacent sacrum, but without joint space narrowing or erosions [18]. Treatment is generally conservative including physical therapy and conservative pain control measures; rarely surgical resection of the lesions is required for refractory cases [18].

### Infectious sacroiliitis

Sacroiliac infection is extremely rare, only accounting for 1–4% of bone and joint infections [23, 24], with a predilection for children and young individuals [24]. This is generally unilateral but bilateral infections have been reported [25]. Musculoskeletal symptoms are essentially indistinguishable from other causes of sacroiliitis: low back and buttock pain as well as posterior thigh pain with difficulty walking on the affected side. *Staphylococcus aureus* is the most frequent organism recovered from synovial or blood specimens in cases of infectious sacroiliitis but *Streptococcus* species, *Escherichia coli,* and *Salmonella* species have also been reported [23, 26].

Brucellosis is a zoonotic infection caused by small, Gram-negative coccobacilli called *Brucella*, of which four to five [27] different species which infect humans [28]. This granulomatous infection is also known as Maltese fever, undulant fever or Mediterranean fever [27]. Infection occurs after contact, ingestion, or inhalation of organisms from infected animals including cattle, goats, and sheep [28]. Although it is endemic to the Mediterranean and Middle East, human brucellosis incidence is low in the United States (0.18 cases/100,000 person years) [28]. There is also a genetic susceptibility to *Brucellosis* infection conferred on the HLA-B39 allele [29]. Approximately 70% of patients with *Brucella* infection experience fever in addition to arthralgias [25]. Arthralgias, myalgias, and back pain often occur with the fever, which is either acute, chronic or relapsing [28].

In addition to other serious systemic manifestations, the skeletal system is one of the most common sites affected by brucellosis in 20–40% of cases [30]. Four studies, which included over 750 patients, reported that between 21 and 55% of individuals infected with *Brucella* experienced bone involvement [31]. Vertebral osteomyelitis is common, particularly in Mediterranean populations [28]; however, the sacroiliac joint is the most commonly reported osteoarticular space for this infection. In one series, sacroiliitis secondary to brucellosis was identified in 26% of 69 proven Brucellosis cases with osteoarticular manifestations [30]. Another review indicates that a quarter to half of all musculoskeletal involvement with brucellosis include the SI joint [24]. Pathogenesis of *Brucella* arthritis is likely from hematogenous spread; septic arthritis is evidenced by recovery of *Brucella* organisms from synovial fluid [32].

Radiographic changes of infectious sacroiliitis begin with extensive erosions and subsequent bony repair, which may involve more than the anterior-inferior synovial part of the joint [5]. Radiograph findings of infectious sacroiliitis are delayed about two weeks [1, 24]. Erosions can be present with joint space enlargement and typical “postage stamp bone surface” [24]. CT has been more sensitive than plain film in finding erosions and joint space widening in infection, and MRI even more so. Though findings are not specific [33], MRI is the imaging modality of choice with fat-suppressed T2-weighted and fluid-sensitive sequences. MRI will demonstrate intra-articular fluid, bone marrow edema, and periarticular involvement, especially during the early phase of the disease. It will demonstrate subchondral sclerosis, erosions and ankylosis in chronic infection setting [34]. Bone scintigraphy has been shown to have high sensitivity, though low specificity for identifying SI infection [24].

### Sacroiliitis and sarcoidosis

Sarcoidosis is a systemic chronic granulomatous disease, which commonly affects the skin, pulmonary and musculoskeletal systems. Racial and familial aggregation of this disease has led to discoveries in support of a genetic association with sarcoidosis, specifically the *BTNL2* gene in the MHC II region on chromosome 6 [35]. Sarcoidosis can affect any organ system–osseous sarcoidosis is well described. One of the rare manifestations of osseous sarcoidosis is involvement of sacroiliac joints. A handful of case reports describe this entity. Although unusual, small cohort studies demonstrate that prevalence of radiographic sacroiliitis in sarcoidosis varies from approximately 6% [36] to 14% [37, 38]. Most sacroiliitis cases of sarcoidosis are unilateral [38], but bilateral cases have been described [39].

Sacroiliac involvement from sarcoidosis may present without a typical inflammatory back pain history [40]. SI joint involvement in sarcoid patients may be due to sarcoid osteitis or granulomatous joint infiltration. Radiographic evidence of sacroiliitis is similar to that of AS, and further imaging and studies are often necessary to elucidate the diagnosis [40]. An FDG-PET scan can be helpful to ascertain involvement [41]. MRI is very sensitive for bony changes in sarcoidosis, and though not specific [40], may aid in choosing a biopsy site. There are rare reports of sarcoidosis coexisting with spondyloarthropathy [36, 42, 43]. Biopsy of the SI joint in sarcoid cases demonstrates non-caseating granulomata in the synovium [44, 45]; however, non-specific inflammatory changes have been described [39]. Even with biopsy changes consistent with sarcoid, tuberculous infection must be ruled out as well.

## Conclusions

Patients with radiographic sacroiliitis and back pain are commonly referred to rheumatologists to rule out AS. One should be aware of other AS mimics and causes of sacroiliitis including Paget disease, DISH, OCI, infections, and sarcoidosis. As depicted in Table 2, there are some features in the pelvic radiographic findings that help distinguish among mimics, however, one must also heavily rely on extra-pelvic radiographic lesions. In addition to the clinical presentation, nuances may hint a clinician to the correct diagnosis. Rheumatologists should be familiar with the imaging differences among these diseases and classic spondylitis findings.

## Additional file

Additional file 1: Timeline of Case Patient. This timeline depicted is the presentation of the case patient with physical exam, imaging, laboratory findings. (DOCX 29 kb)

## Abbreviations

AP: Anterior posterior
AS: Ankylosing spondylitis
CT: Computer tomography
DISH: Diffuse idiopathic skeletal hyperostosis
FABER: Flexion-abduction-external-rotation
MRI: Magnetic resonance imaging
OCI: Osteiitis condensans ilii
SI: Sacroiliac
