# Supplementary material for: Sacroiliitis mimics: a case report and review of the literature
Source: BMC Musculoskelet Disord. 2017 Apr 22;18:170. doi: 10.1186/s12891-017-1525-1 (PMC5401462; doi:10.1186/s12891-017-1525-1)
Supplement: Supplementary file 1 [file 12891_2017_1525_MOESM1_ESM.docx]

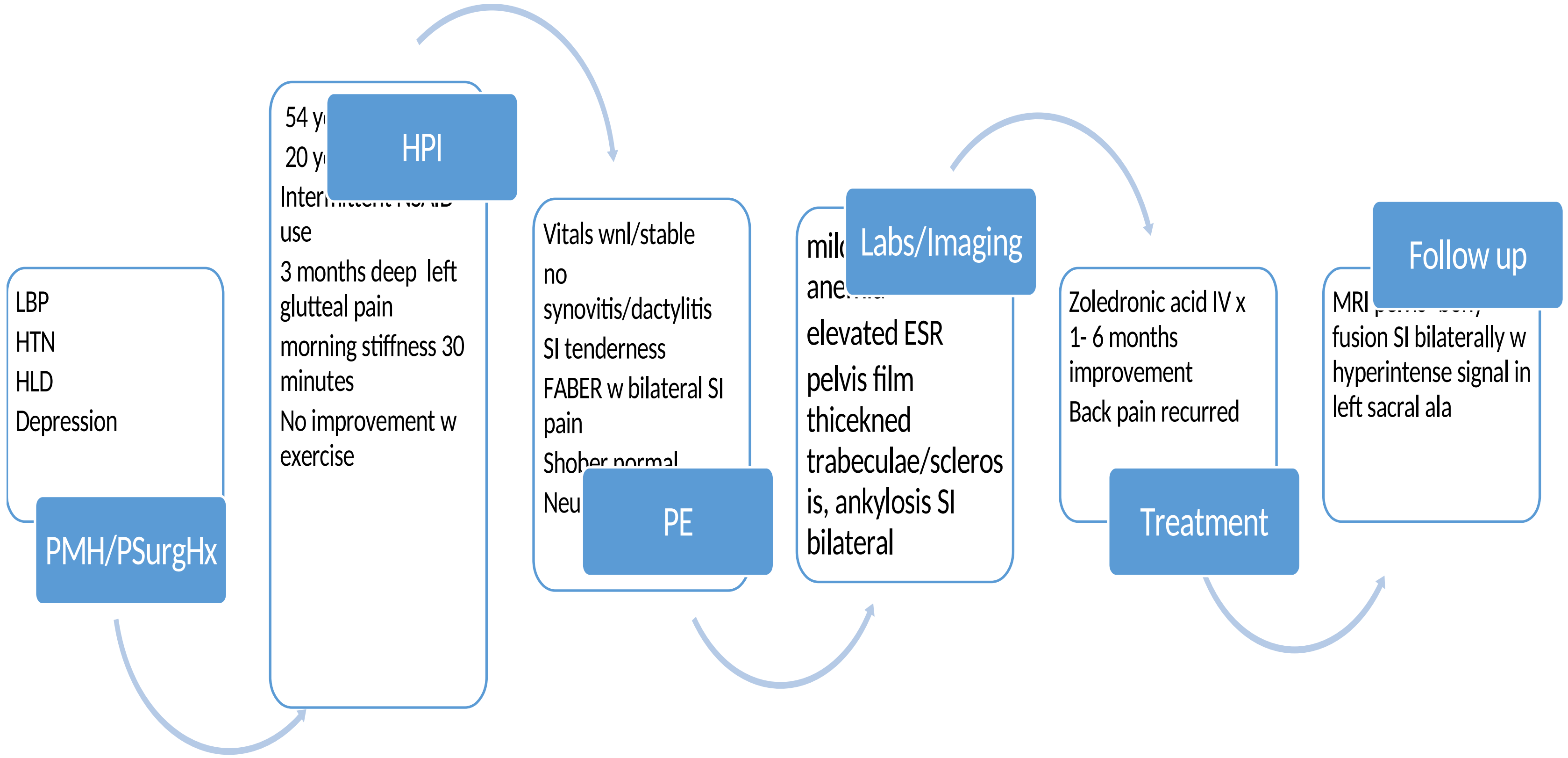
LBP
HTN
HLD
Depression
PMH/PSurgHx
HPI
use
3 months deep left glutteal pain
morning stiffness 30 minutes
No improvement w exercise
Vitals wnl/stable
no synovitis/dactylitis
SI tenderness
FABER w bilateral SI pain
Shober normal
PE
Labs/Imaging
elevated ESR
pelvis film thickend trabeculae/sclerosis, ankylosis SI bilateral
Zoledronic acid IV x 1- 6 months improvement
Back pain recurred
Treatment
Follow up
MRI
fusion SI bilaterally w hyperintense signal in left sacral ala
